# Mixed methods usability evaluation of an assistive wearable robotic hand orthosis for people with spinal cord injury

**DOI:** 10.1186/s12984-023-01284-8

**Published:** 2023-12-01

**Authors:** Jan Dittli, Jan T. Meyer, Jessica Gantenbein, Tobias Bützer, Raffaele Ranzani, Anita Linke, Armin Curt, Roger Gassert, Olivier Lambercy

**Affiliations:** 1 Rehabilitation Engineering Laboratory, Department of Health Sciences and Technology, ETH Zurich, Lengghalde 5, 8008 Zurich, Switzerland; 2 Spinal Cord Injury Center, Balgrist University Hospital, Forchstrasse 340, 8008 Zurich, Switzerland; 3 Singapore-ETH Centre, Future Health Technologies Programme, CREATE campus, 1 Create Way, #06-01 CREATE Tower, 138602 Singapore, Singapore

**Keywords:** Usability, Robotic hand orthosis, Mixed methods, Wearable robots

## Abstract

**Background:**

Robotic hand orthoses (RHO) aim to provide grasp assistance for people with sensorimotor hand impairment during daily tasks. Many of such devices have been shown to bring a functional benefit to the user. However, assessing functional benefit is not sufficient to evaluate the usability of such technologies for daily life application. A comprehensive and structured evaluation of device usability not only focusing on effectiveness but also efficiency and satisfaction is required, yet often falls short in existing literature. Mixed methods evaluations, i.e., assessing a combination of quantitative and qualitative measures, allow to obtain a more holistic picture of all relevant aspects of device usability. Considering these aspects already in early development stages allows to identify design issues and generate generalizable benchmarks for future developments.

**Methods:**

We evaluated the short-term usability of the RELab tenoexo, a RHO for hand function assistance, in 15 users with tetraplegia after a spinal cord injury through a comprehensive mixed methods approach. We collected quantitative data using the Action Research Arm Test (ARAT), the System Usability Scale (SUS), and timed tasks such as the donning process. In addition, qualitative data were collected through semi-structured interviews and user observations, and analyzed with a thematic analysis to enhance the usability evaluation. All insights were attributed and discussed in relation to specifically defined usability attributes such as comfort, ease of use, functional benefit, and safety.

**Results:**

The RELab tenoexo provided an immediate functional benefit to the users, resulting in a mean improvement of the ARAT score by 5.8 points and peaking at 15 points improvement for one user (clinically important difference: 5.7 points). The mean SUS rating of 60.6 represents an adequate usability, however, indicating that especially the RHO donning (average task time = 295 s) was perceived as too long and cumbersome. The participants were generally very satisfied with the ergonomics (size, dimensions, fit) of the RHO. Enhancing the ease of use, specifically in donning, increasing the provided grasping force, as well as the availability of tailoring options and customization were identified as main improvement areas to promote RHO usability.

**Conclusion:**

The short-term usability of the RELab tenoexo was thoroughly evaluated with a mixed methods approach, which generated valuable data to improve the RHO in future iterations. In addition, learnings that might be transferable to the evaluation and design of other RHO were generated, which have the potential to increase the daily life applicability and acceptance of similar technologies.

**Supplementary Information:**

The online version contains supplementary material available at 10.1186/s12984-023-01284-8.

## Introduction

Over the past decade, a diverse set of wearable robotic hand orthoses (RHO) for grasping assistance have been developed and tested in people with sensorimotor hand impairment. RHO designs range from fully portable to stationary devices and include various actuation and control principles to match the needs of their target users [1–4]. Depending on the variety of integrated functions, the design of the technological solutions widely differ in complexity, ranging from textile-based soft robotics with, e.g., pneumatic actuators to more rigid devices with 3D printed or carbon-fiber-based structures. While several promising research prototypes have shown their potential to support hand function and assist people with sensorimotor hand impairment, only very few have been made available for the target users as certified products [2]. One major factor limiting the translation from wearable robotic device prototypes to products has been frequently reported as the unsatisfactory daily life usability of the novel, increasingly complex rehabilitation technologies [5, 6].

To address this adoption barrier preemptively, a deliberate focus on user-centered design and, specifically, early in-depth evaluation of device usability could help improve the RHO functionality and increase the technology acceptance for daily life [7]. Usability evaluation aims to assess the extent to which a technical solution fulfills the user needs and requirements that originate from the targeted problem. In the case of RHO, this target problem is the limited ability to perform activities of daily living (ADL) due to disabilities, leading to reduced quality of life. The effectiveness of RHO in solving this problem has been investigated in numerous short-term, single-session usability studies [8–13]. Such short-term usability evaluations aim to showcase the immediate functional benefit provided by a technology, as an indicator for their initial acceptance. This functional benefit was mainly assessed by adopting quantitative, clinically validated measures such as the Toronto Rehabilitation Institute Hand Function Test (TRI-HFT) [8, 14] or the Jebsen-Taylor Hand Function Test (JTHFT) [9, 10]. Similarly, the RELab tenoexo, a fully wearable, compact RHO that can assist full hand grasping in people with tetraplegia after spinal cord injuries (SCI), proved its effectiveness in terms of an immediate functional benefit measured by the Action Research Arm Test (ARAT) [15]. However, overall usability is not only determined by effectiveness but also user satisfaction and usage efficiency [16]. Satisfaction and efficiency, which both are often easier to assess qualitatively, were not investigated in these studies and generally fall short in RHO evaluation studies [7]. While functional assessments are often complemented by custom-made usability questionnaires to quantify the subjective opinions, a structured evaluation of user needs and pain points, or the assessment of RHO design-specific questions with qualitative methods such as semi-structured interviews or focus groups is often lacking [17]. It thus remains mostly unclear whether the RELab tenoexo and RHO in general fulfill overall expectations and needs by users to achieve acceptance for daily use. We hypothesize that a comprehensive, mixed methods evaluation, i.e., a combination of quantitative and qualitative measures, would provide more holistic insights on all relevant aspects of RHO usability [18, 19]. While quantitative metrics allow for an outside comparison to existing benchmarks or across iterations of the same device, qualitative findings may provide further insights to interpret these results and to identify limiting factors that could influence the daily use of RHO [20]. With qualitative evaluation, it is simpler to identify residual design issues and obtain feedback from users on necessary design changes that may need to be addressed in the following design iteration.

In this study, an in-depth evaluation of the short-term usability of the RELab tenoexo is presented using a comprehensive mixed methods approach. We conducted a battery of usability assessments, including hand function tests, RHO-specific performance metrics, a range of usability questionnaires, as well as semi-structured interviews to investigate the usability of the RHO in terms of effectiveness, efficiency, and satisfaction. In order to make the rather vague usability dimensions more understandable and practicable in the specific case of RHO short-term usability, we present a list of specific usability attributes to investigate, identifying the key strengths and weaknesses of the RHO. A total of 15 users with tetraplegia tested the RELab tenoexo by completing functional tasks relevant to daily life in a laboratory environment and provided extensive feedback on their experience with using an RHO for the first time. Participants with different levels of hand impairment were included to investigate the match between the available technology and the user needs to further refine the target population of the RELab tenoexo. The gained insights allowed to underline essential areas for improving the usability of the RELab tenoexo. The presented evaluation approach could be generalized to the user-centered design of RHO, to potentially improve their daily life applicability and technology uptake by end users.

## Methods

### Robotic hand orthosis

**Fig. 1:**
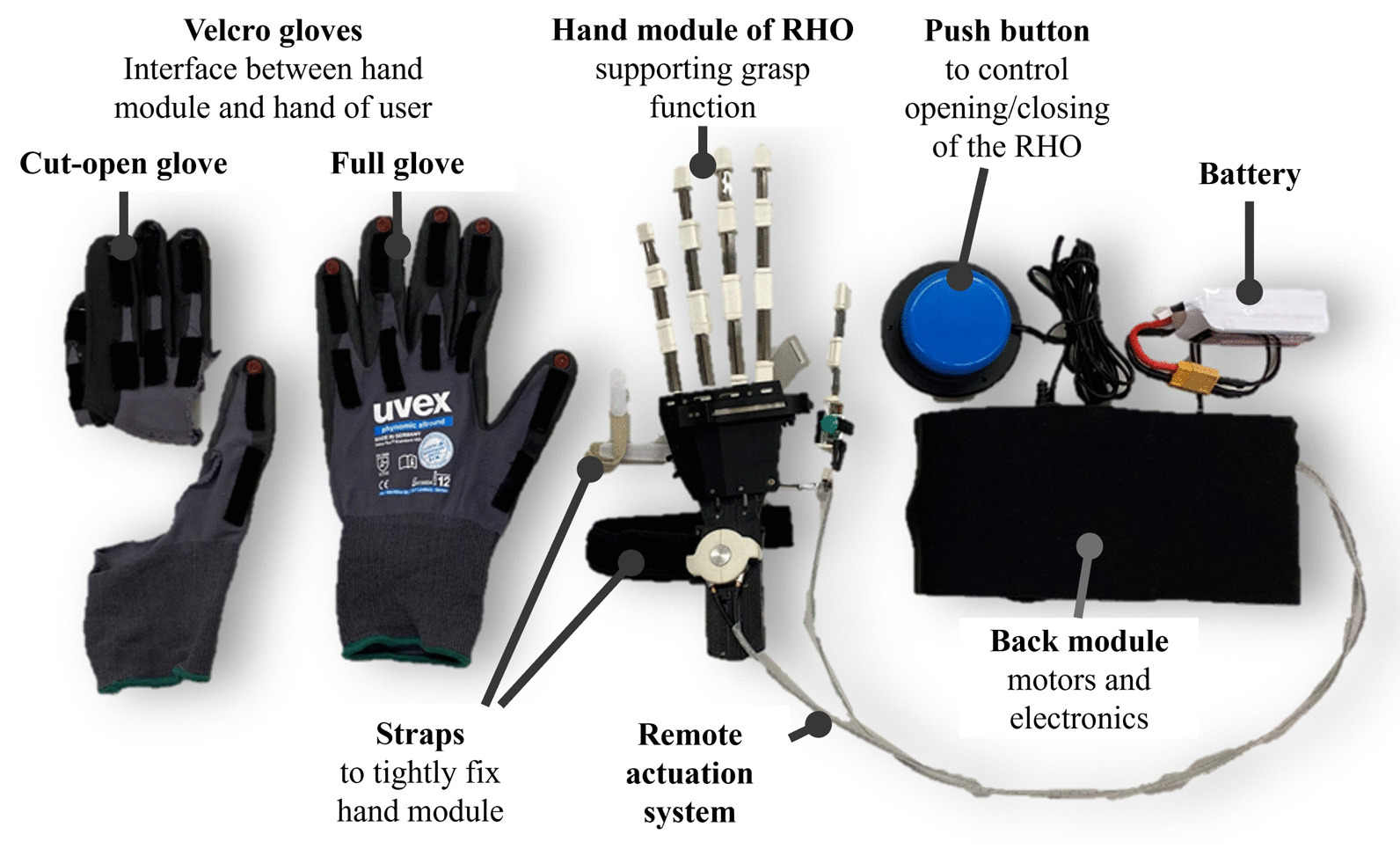
Overview of the RELab tenoexo RHO system: The fully wearable and portable RHO supports grasp function in people with sensorimotor hand impairment. The hand module is actuated via a remote actuation system. Battery, motors, and electronics are placed in the back module. A full or cut-open glove and straps allow to fix the hand module on a user’s hand. A push button is used to trigger opening/closing of the RELab tenoexo

In this study, the short-term usability of the RELab tenoexo, a fully wearable and portable RHO, was evaluated (Fig. 1). The RELab tenoexo can support grasp function in people with sensorimotor hand impairment through active finger flexion and extension assistance [15]. The main components of the RELab tenoexo are a hand module (weight 120 g), which is attached to the user’s hand to support grasping, and a back module (weight 492 g), containing the electronics, motors, and battery. The back module can be worn as a backpack or mounted on the backrest of a wheelchair. Two Bowden cable-based remote actuation systems (RAS) transmit the force from the motors to the hand module [21]. One RAS actuates thumb flexion and extension, while the second one actuates flexion and extension of index, middle, ring, and little finger combined. The thumb can be moved to opposition using a manual slider to allow performing the most relevant grasp types necessary for daily tasks. The RELab tenoexo provides sufficient force to grasp objects weighing up to 0.5 kg (i.e., approx. 5N per finger) and closes and opens within 1 s. In order to mount the device on the hand, a dedicated textile glove (i.e., full glove or cut-open for simpler donning), which contains Velcro on the dorsal side of each digit, is donned to the user. This Velcro can then be attached to its counterparts aligned on the palmar side of the hand module. Textile straps around the wrist and the palm can be tightened to securely attach the hand module to the user’s hand and forearm. In this configuration, the hand module acts as a passive wrist orthosis fixing the wrist position in a slightly extended functional position.

The current physical attachment solution is intended for assisted use, meaning that the target users are not expected to be able to don the device independently. For this study, two hand module sizes were available, dimensioned according to the hand sizes of an average adult male and female. For each size, a left-handed and a right-handed version were available. While different intention detection strategies could be used to trigger opening/closing of the RHO [4], in this study a large-diameter push button was used, which was connected to the back module via a cable. Pushing the button triggered the RAS to apply the maximum force in closing direction. The opening force was limited so that it was sufficient to open the fingers of the participants to a slightly extended position but not overstretching the fingers.

### Recruitment

Participants were recruited at the Rehabilitation Engineering Laboratory of ETH Zurich and at the Spinal Cord Injury Center of Balgrist University Hospital Zurich by therapist referral or through a participant database. All participants gave written informed consent, and all experimental procedures were approved by the ethics committee of ETH Zurich (2018-N-90). Individuals with sensorimotor hand impairment after SCI who were above 18 years of age, able to give informed consent, and understand the tasks involved in the trial were eligible to participate in the study. Major depression or deficits in cognition, communication, comprehension, or memory were defined as exclusion criteria. No further inclusion or exclusion criteria based on upper-limb function where defined to explore the potential target user group of the RELab tenoexo.

### Usability attributes and outcome measures

The objective of this work was to administer a mixed method approach to obtain a holistic picture of the short-term usability of the RELab tenoexo to assist hand function in people with SCI. Previous analyses of usability evaluation practices in wearable robotics showed that most studies focus on specific usability attributes rather than evaluating usability in terms of the dimensions effectiveness, efficiency, and satisfaction [4, 7, 22]. Combining these insights with other works that highlighted the most relevant usability attributes for RHO target users [23, 24], we defined and specifically investigated the following “core usability attributes” in this study:

- Functional benefit: The immediate positive effect on the user’s functional capabilities gained from the RHO (e.g., ability to perform tasks with an RHO that can not be performed without support)
- Device functionality: The quality, or range of functions provided by the RHO (e.g., mechanical properties, range of motion, degrees of freedom, number of provided grasp types)
- Ease of use and practicability: The degree to which using the RHO is free of unnecessary effort (e.g., absence of mobility constraints from using the device)
- Comfort and ergonomics: The extent to which the use of the RHO does not induce pain, unnecessary constraint, or unpleasant feeling, and the degree of kinematic compatibility between the user and the RHO (e.g., no pressure marks or pain from using the RHO)
- Safety: The condition of being safe; the extent to which the use of a system is free from danger or risk of injury

Quantitative measures were selected to evaluate and cover all core attributes taking inspiration from an online database [22] (Table 1). In addition, qualitative data from user statements in semi-structured interviews and user observations on the active RHO use were analyzed to complement, confirm, and extend the insights from the quantitative outcomes.

#### Action Research Arm Test

The Action Research Arm Test (ARAT) was selected to evaluate the usability attributes *functional benefit* and *device functionality*. The ARAT is a standardized, observational assessment of upper limb function consisting of 19 objects to be manipulated using a range of grasp types. The objects are assigned to the subscales “Grasp”, “Grip”, “Pinch”, and “Gross movement”. The performance of manipulation is rated on a 4-point scale (0: no movement, 3: movement performed normally), resulting in a score between 0 (min) and 57 (max) [25, 26]. The assessment was administered once with and once without the RHO to objectively evaluate its immediate functional benefit for the user without any prior training except for a short tryout.

#### Donning and wearing times

The donning was performed unhurried and assisted by a study coordinator or a family member accompanying the participant, representing a realistic usage in daily life. The donning time was clocked from the first contact of any RHO component with the participant’s hand until the fixation of the last strap after verifying a comfortable fit with the participant. The core attributes *ease of use* and *practicability* were evaluated with this objective, quantitative measure. The personalized placement of the push button was not included in the donning time as this continuously changed during the sessions based on usage experiences and preferences. In addition, the total active wearing time of the RHO (i.e., from donned and ready to use to doffing) was also analyzed in order to relate if and how a prolonged use would affect the *comfort and ergonomics*.

Both the donning and the wearing times were extracted from video recordings of the study. No parts of the study were actively timed onsite, meaning that the participants were given all the time they needed and desired for each task.

#### System Usability Scale

To complement the quantitative dataset with subjective measures, usability questionnaires were administered. As a standardized benchmark, the System Usability Scale (SUS) was used. The SUS is a widely accepted and validated usability questionnaire consisting of 10 statements (also called “items”) rated on a 5-point scale (1: strongly disagree, 5: strongly agree) [27]. The items are phrased positively and negatively in an alternating order, where odd items are phrased positively (i.e., rating of 5 most positive) and even items are phrased negatively (i.e., rating of 5 most negative). A validated German translation of the SUS was used [28]. Each individual SUS item was carefully analyzed and ascribed to the attributes *functional benefit*, *ease of use*, *device functionality*, and *safety* as perceived by the user. For reference in this paper, the SUS items were labelled Q1 to Q10 (Table 3).

#### Custom usability questionnaire

In addition to the SUS, a custom usability questionnaire (CUQ) consisting of 10 items rated in a comparable 5-point Likert scale (1: strongly disagree, 5: strongly agree) was administered (Table 4). The CUQ was introduced to complement the standardized SUS with items that specifically assessed core usability attributes that the ARAT and SUS did not sufficiently cover (e.g., *comfort* and *ergonomics*), as well as to provide a more RHO-specific understanding of technical attributes such as generated force and movement speed. The CUQ items were labelled Q11 to Q20 for reference in this paper.

**Table 1:** Assignment of quantitative outcome measures and specific subquestions/statements (Q) to core usability attributes

Attribute	ARAT score	SUS item	CUQ item	Donning time, wearing time	Adverse events, technical issues
Functional benefit	X	Q1	Q11, Q12, Q13		
Device functionality	X	Q1, Q5, Q6	Q15, Q16, Q17		X
Ease of use & practicability		Q3, Q4, Q8	Q14	X	
Comfort & ergonomics	X		Q18, Q19, Q20	X	X
Safety		Q9			X

#### Qualitative evaluation

For the qualitative evaluation of the RHO, a semi-structured interview was administered to better understand the perceived usability in the words of the users. The interview questions were defined based on the research question and topics the study coordinators wanted to address and their formulation followed basic guidelines for open-ended interview questions, such as omitting yes/no answers or leading questions [29]. The full list of interview questions (translated from German) can be found in Additional file 1: File 1. At the start of each session, the participants were asked about their current rehabilitation status (7 questions, Q21–Q27) and their unmet needs or wishes (Q28–Q30). After the RHO testing and the completion of the questionnaires mentioned above, the interview was continued to collect as much direct feedback on the RHO as possible (Q31–Q38). The leading study coordinator took care of the moderation and study protocol progression. Additionally, user statements during use (i.e., spontaneous thinking aloud) and specific user observations during RHO use were noted by a second study coordinator. The qualitative feedback was expected to augment the quantitative data and potentially generate additional insights.

#### Adverse events and technical issues

Potential adverse events and technical issues occurring during the sessions were recorded as an additional measure of the *safety* and *device functionality* of the RHO.

### Study protocol and setup

**Fig. 2:**
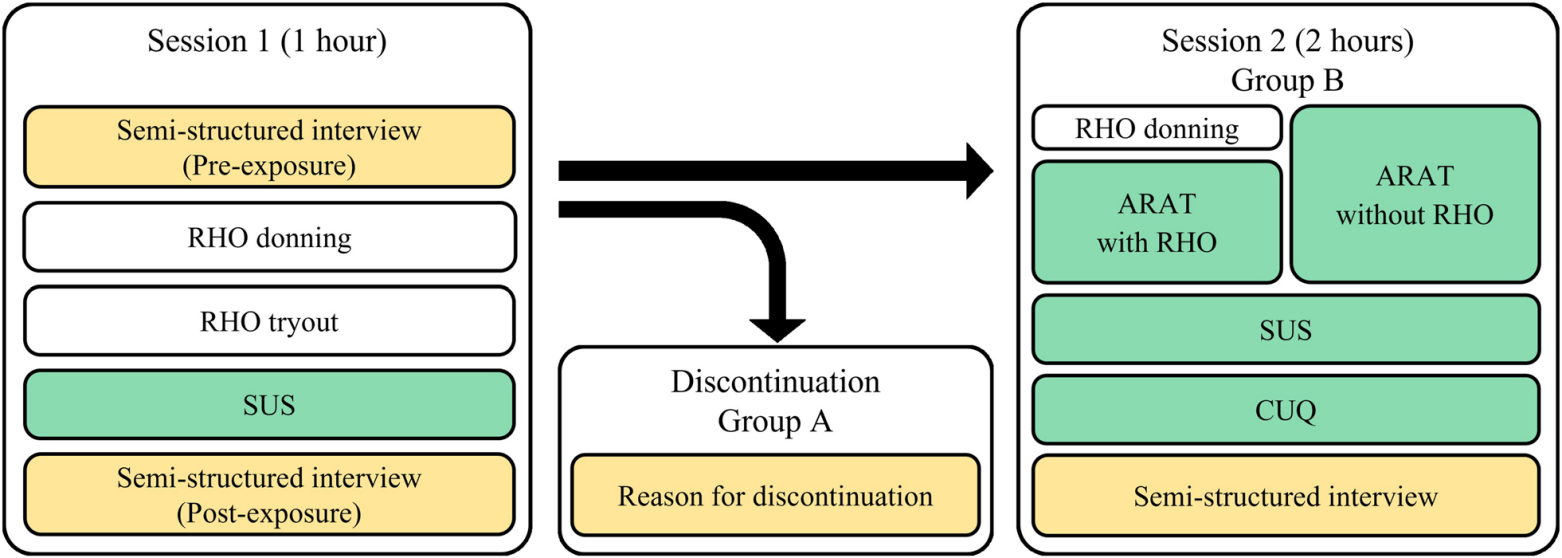
Study protocol: Quantitative (green) and qualitative (yellow) measures were used to assess the short-term usability of the RELab tenoexo in two sessions. Session 1 consisted of a tryout of the RHO, interviews, and a standardized questionnaire (i.e., SUS). Group A discontinued the study after session 1, while group B attended an extended session 2. In session 2, the participants performed the ARAT with and without RELab tenoexo (randomized order) and provided additional feedback (i.e., in interviews, SUS, and CUQ)

The usability study consisted of two sessions (Fig. 2). Session 1 served as a screening session to (i) identify participant characteristics and needs (e.g., challenges in daily life not specific to RHO), (ii) try out the RELab tenoexo (i.e., donning, grasping different objects such as a water bottle, a pen, or a cup), and (iii) collect user feedback on the first impression of the RHO through the semi-structured interviews and the SUS questionnaire.

After session 1, a decision was made together with the participants to either proceed to study session 2 or discontinue the study based on the user experience as well as expected benefit from the RHO for the individual level of impairment. Those participants that discontinued after session 1 were assigned to group A, and those that continued with the study were assigned to group B. The reasons for discontinuation were recorded. Group B was then invited to attend the extended session 2 at least one week after session 1. In session 2, the SUS and interviews from session 1 were repeated. In addition, the immediate effect on the participant’s hand function when using the RHO was assessed and compared to the unassisted condition without RHO using the ARAT. After the ARAT, more detailed user feedback on the RELab tenoexo use was collected, including the CUQ questionnaire.

**Fig. 3:**
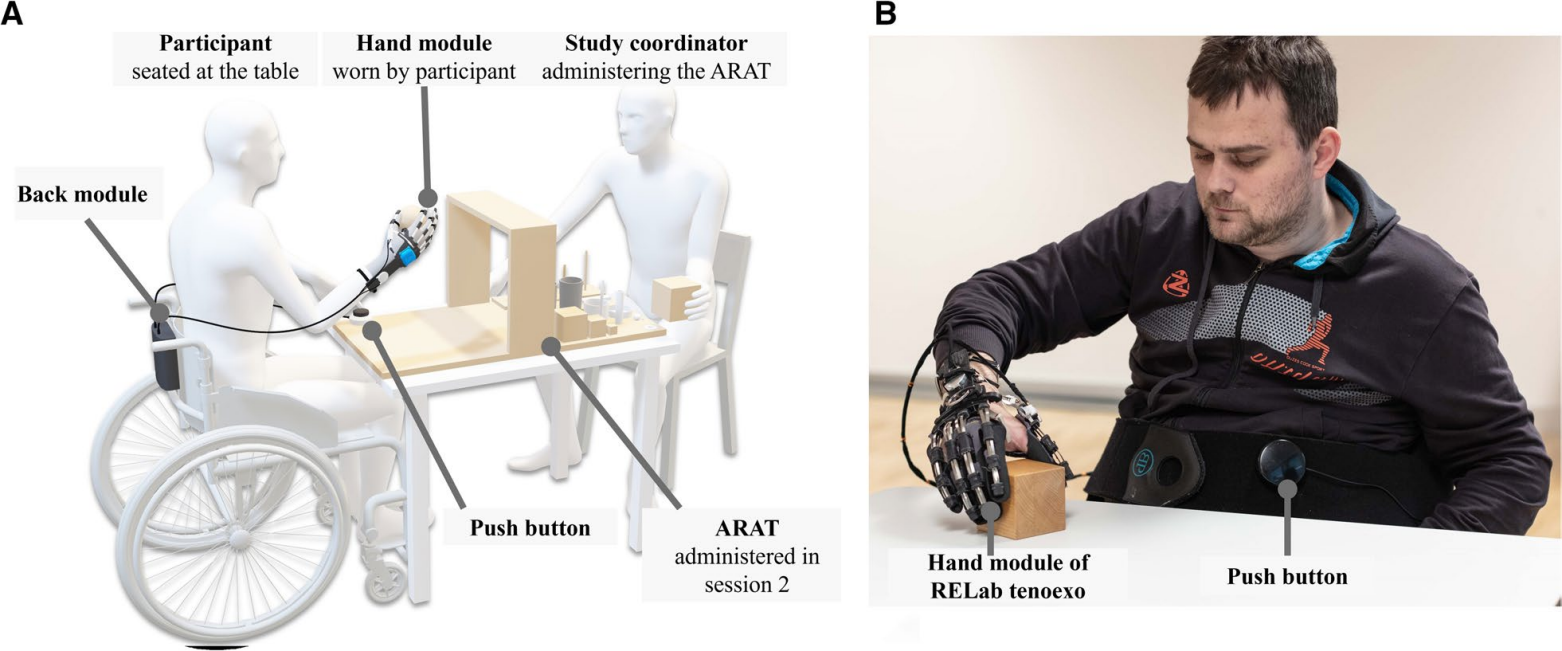
Study setup: **A** The participant wearing the hand module of the RELab tenoexo was comfortably seated at a table to perform session 2 of the study. The back module was placed on the backrest of the wheelchair or the table. A study coordinator sitting opposite of the participant guided through the entire mixed methods protocol and administered the hand function test ARAT. **B** The participants controlled the RELab tenoexo via a push button to grasp different objects of the ARAT

For the administration of the ARAT, the participants were seated at a table that was adjusted to a comfortable height (Fig. 3). The RELab tenoexo was donned and used on the preferred hand of the participant, which in most cases was the more severely affected hand. The participants either wore the full glove or the cut-open glove (only covering the fingers but not the palm) to don the RHO. They were allowed to manually adjust the thumb position via the opposition mechanism according to their preference independently or, if needed, with the support of a study coordinator. The back module was mounted on the backrest of the wheelchair or placed on the table. The push button was placed on the table, the user’s body, or the armrest of the wheelchair according to the participant’s preference. A study coordinator (JD, JTM, or JG) was seated vis-a-vis the participant during the entire session to guide through the protocol. Both sessions were video recorded and carefully observed by a second study coordinator responsible for documenting the study. All study parts were conducted in a controlled setting in a clinic or a research laboratory. The recruitment for this study was coordinated and guided by occupational therapists, and the study procedure was led by a mix of health scientists and mechanical engineers.

### Data analysis and statistics

All data collected within sessions 1 and 2, as well as the reasons for discontinuation, were included in the analysis.

#### Quantitative analysis

The ARAT scores were rated by an independent, trained occupational therapist based on the video recordings after conclusion of all study sessions to ensure validity and consistency across all participants. This therapist was not part of the study team and was therefore blinded to the rest of the study. The total SUS score was calculated using the original equation by Brooke et al. [27]:

$$\begin{aligned} SUS_{total,original}= \left(\sum _{i=1,3,5,7,9}(Qi-1)+\sum _{i=2,4,6,8,10}(5-Qi)\right)*2.5 \end{aligned}$$

which results in a score between 0 (min) and 100 (max). For easier visual comparability between the individual SUS item ratings (*Qi*), we inverted the statements of negatively phrased items (*Q*2, *Q*4, *Q*6, *Q*8, *Q*10) in Table 3 to make all statements positive and adapted the scoring accordingly. Similar to the SUS, a total score of the CUQ items was calculated as follows:

$$\begin{aligned} CUQ_{total}=\sum _{i=11}^{20}Q_i*2.5 \end{aligned}$$

also resulting in a total score ranging from 0 to 100.

The statistical analysis of the data was done using Python [30] and SciPy [31]. Due to the small sample size, the non-parametric Wilcoxon signed-rank test was performed to determine differences between the conditions without and with the RHO as well as the change in the SUS score between sessions 1 and 2. The Z-statistic value of the Wilcoxon signed-rank test and the p-value were reported. Pearson correlation was used to investigate potential correlations between individual changes in ARAT score and questionnaire ratings (i.e., perceived benefit, usability, ease of use).

#### Qualitative analysis

The collected qualitative data, from notes taken during the sessions and from the recorded video material, was thematically analyzed according to recommended guidelines by three authors (JD, JTM, JG) [32]. All collected statements, including data related to them, were grouped according to the core usability attributes. Additional attributes were introduced when statements covered usability aspects other than the core attributes. The three authors (JD, JTM, JG) discussed the assignment of the statements and outcome measures to attributes until a consensus was reached.

## Results

### Participants

A total of 15 participants completed study session 1, and five moved forward to complete session 2 (group B, mean age 45.6 (standard deviation, *SD* = 19.4) years, 5 male). The detailed participant characteristics and individual reasons for discontinuation of group A can be found in Table 2. Common reasons for discontinuation were: insufficient proximal arm function to complete the ARAT (N = 4), loss of interest in the study (N = 4), and unavailability of appropriate device size (N = 3). Clinical participant characteristics (neurological injury level on the right R/left L motor tract, type of SCI, AIS, GRASSP score R/L) were determined by the clinical personnel of the recruiting hospital prior to the study. All participants were previously naive to the tested RHO but used passive assistive devices before in their daily life (e.g., wheelchair gloves, passive orthoses, wheelchair). Six participants had experience with robot-assisted rehabilitation, such as the Lokomat and ArmeoSpring (Hocoma AG, Volketswil, CH) or the FLOAT (Reha-Stim MedTech, Inc., Schlieren, CH).

**Table 2:** Participant characteristics

Participant characteristics									Reasons for discontinuation					
Participant ID	Motor level R/L	Type of spinal cord injury	Time since injury (months)	AIS	GRASSP R/L (score 0–116)	Dominant/tested hand	Gender	Age (years)	Proximal arm function insufficient	Appropriate size of RHO not available	Extension force by RHO insufficient	Fear of losing tenodesis grasp	Participant lost interest	Participant not available anymore
P1	C5/C5	Incomplete	4	C	15/11	R/R	M	65	X	X			X	
P2	C5/C5	Incomplete	1	D	0/5	R/R	M	36	X					
P3	C4/C5	Incomplete	5	D	29/69	R/R	M	37	X		X			
P4	C3/C2	Incomplete	8	C	16/28	R/R	M	30		X				X
P5	C5/C2	Incomplete	6	C	7/2	R/R	M	81		X			X	
P6	C7/C6	Incomplete	85	C	39/39	L/R	F	38			X	X		
P7	C3/C3	Incomplete	4	C	13/12	R/R	M	28					X	
P8	C3/C5	Incomplete	12	C	44/21	R/R	M	51	X					
P9	N.D./C7	Incomplete	9	N.D.	5/41	R/L	M	43						X
P10	C7/C7	Incomplete	348	C	N.A.	R/L	M	58					X	
P11*	C6/C6	Complete	57	A	N.A.	R/R	M	30	Continued					
P12*	C6/C6	Complete	6	A	16/21	L/R	M	29	Continued					
P13*	C8/C8	Incomplete	8	D	64/63	R/R	M	51	Continued					
P14*	N.D./N.D.	Other$^{\bullet }$	78	C	20/42	L/R	M	81	Continued					
P15*	C7/C7	Complete	82	A	46/44	R/R	M	37	Continued					
							Total		4	3	2	1	4	2

*Group B: participated in both sessions, $^{\bullet }$Other: Tetraparesis, N.A.: not available, N.D.: not defined

### Outcome measures

#### Action Research Arm Test

**Fig. 4:**
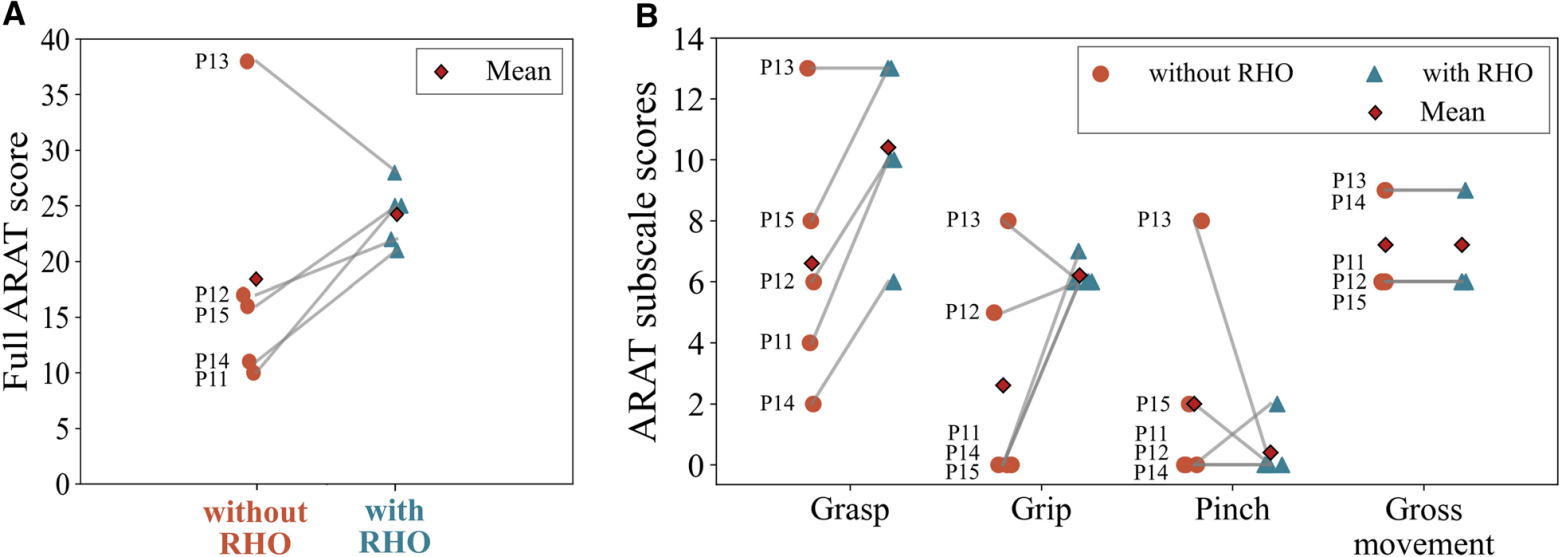
Results of the ARAT administered within study session 2 (group B): **A** an overall mean improvement of 5.8 points was observed when using the RHO to complete the ARAT. Four out of five participants improved their overall ARAT score. **B** Improvements in the ARAT subscales “Grasp” and “Grip” were observed. The scores in the subscale “Pinch” decreased for most participants. The “Gross movement” scores remained unchanged

For group B, the total ARAT scores with and without RHO are presented in Fig. 4A, and the individual subscales are presented in Fig. 4B. With the assistance of the RHO, the ARAT scores of four participants improved (P11*, P12*, P14*, P15*) by + 15, + 5, + 10, and + 9 points, respectively (clinically important difference: 5.7 points [33]). Participant P13* achieved the highest ARAT score among all participants without RHO and performed worse (− 10 points) with the RHO. With the RHO, all participants achieved a similar total score around a mean of 24.2 (*SD* = 2.5) points, and the mean change of the ARAT score across all participants was +5.8 points compared to the condition without RHO (Z = 3.5, *p* = 0.28). For the four participants who improved their ARAT score when assisted (mean +9.75 points), the largest increase in hand function by the RHO was achieved in the “Grasp” and “Grip” subscales. The scores in the subscale “Pinch” decreased for most participants. The “Gross movement” scores remained unchanged. Based on observations from the study coordinators, the main limiting factors for the performance scores were, e.g., insufficient proximal arm function to accurately position the hand with respect to the object, lack of proximal strength and grasping force to lift heavier objects, and missing dexterity to grasp smaller objects (e.g., inability to move single digits).

#### Donning and wearing time

On average, donning the RHO took 4 min 55 s, whereas individual times ranged from 1 min 43 s to 9 min 13 s. The main challenges leading to prolonged donning times with the study participants were high tone or tenodesis grasp hindering finger extension while trying to slip into the glove, inappropriate glove sizes (i.e., fingers slipping out easily or challenges to insert them), or the need for additional fixation such as tape or textile straps if the Velcro became loose.

The average RHO wearing time was 19 min 45 s for session 1 and 37 min 15 s for session 2. The maximum continuous wearing was 54 min (P13* in session 2).

#### System Usability Scale

Mean SUS scores are presented in Table 3. Across all participants, a mean SUS score of 60.6 (*SD* = 17.7) out of 100 points was achieved, indicating an “ok” to “good” usability based on the benchmark by Bangor et al. [34]. The mean score in group B decreased by 6.9 (Z = 6.0, *p* = 0.68) from session 1 to session 2. Further, the achieved overall SUS scores converged in session 2, as reflected by a smaller standard deviation (*SD* = 8.9) compared to session 1 (*SD* = 15.2). The inversion of the even SUS items to simplify the visual comparison is indicated with the parentheses (e.g., “did not”) in Table 3. The SUS was conducted in the original, alternating format with the participants.

**Table 3:** Results from the System Usability Scale

Item no.	Item	Session	Rating group A Mean (SD)	Rating group B Mean (SD)	Rating all Mean (SD)
*Q*1	I think that I would like to use this system frequently.	1	3.0 (1.4)	3.3 (1.5)	3.1 (1.4)
		2	–	3.2 (1.2)	–
$Q2^\circ$	I *(did not)* find the system unnecessarily complex.	1	3.0 (0.7)	4.3 (0.8)	3.4 (0.9)
		2	–	3.6 (0.8)	–
*Q*3	I thought the system was easy to use.	1	3.0 (1.2)	3.3 (1.1)	3.1 (1.1)
		2	–	3.6 (1.0)	–
$Q4^\circ$	I *(do not)* think I would need the support of a technical person to be able to use this system.	1	2.8 (1.2)	2.3 (1.6)	2.6 (1.4)
		2	–	2.2 (1.5)	–
*Q*5	I found the various functions in this system were well integrated.	1	3.4 (1.0)	4.3 (0.8)	3.7 (1.0)
		2	–	3.2 (1.5)	–
$Q6^\circ$	I *(did not)* think there was too much inconsistency in this system.	1	3.3 (0.8)	4.5 (0.5)	3.7 (0.9)
		2	–	4.0 (0.6)	–
*Q*7	I would imagine that most people would learn to use this system very quickly.	1	4.0 (0.9)	4.5 (0.5)	4.2 (0.9)
		2	–	3.8 (1.0)	-
$Q8^\circ$	I *(did not)* find the system very cumbersome to use.	1	2.9 (1.1)	3.5 (1.1)	3.1 (1.1)
		2	–	3.0 (0.9)	–
*Q*9	I felt very confident using the system.	1	3.3 (1.2)	4.3 (0.4)	3.6 (1.1)
		2	–	4.2 (0.4)	–
$Q10^\circ$	I *(did not)* need to learn a lot of things before I could get going with this system.	1	3.9 (1.0)	3.8 (1.3)	3.8 (1.1)
		2	–	4.2 (1.2)	–
		1	56.7 (17.2)	69.4 (15.2)	60.6 (17.7)
	Total score $SUS_{total}$	2	–	62.5 (8.9)	–

The statements for the even items are inverted to make all points positive (indicated with the parentheses) and the scoring for these items was adapted accordingly

Mean and standard deviation for each item rating and the overall SUS scores are reported for group A (P1–P10), for group B (P11*–P15*) and across all participants (P1–P15*). Item ratings range from 1 (completely disagree) to 5 (completely agree) and total scores range from 0 to 100, where a higher value corresponds to better usability. (*: Group B; $^\circ$: inverted item; SD: standard deviation)

#### Custom usability questionnaire

The mean total CUQ score was 63.5 (*SD* = 16.0) out of 100 points. Individual item ratings from the CUQ and individual results by participant are shown in Table 4. The highest rated RHO-specific items were concerning the weight and the speed of the assisted movements (*M* = 4.4, *SD* = 0.5, and *M* = 4.2, *SD* = 0.7, respectively). The lowest rated usability item was the ease of donning and doffing (*M* = 2.4, *SD* = 0.8). The subjective perception of performance significantly correlated with the respective change in ARAT score (Q11: *r* = 0.99, *p* < 0.01; Q12: *r* =.91, *p* = 0.03; Q13: *r* =.98, *p* < 0.01) on the individual participant level.

**Table 4:** Results from the Custom Usability Questionnaire

Item no.	Item	Score P11*–15* Mean (SD)	P11*	P12*	P13*	P14*	P15*
Q11	With the RHO, performing the tasks is easier.	3.4 (1.4)	5	3	1	4	4
Q12	The RHO helps me to perform tasks quicker.	3.4 (1.4)	5	4	1	3	4
Q13	The RHO gives me more control of my hand activity.	3.6 (1.4)	5	4	1	4	4
Q14	The RHO is easy to don and doff.	2.4 (0.8)	3	3	1	2	3
Q15	The RHO generates sufficient force while closing.	3.0 (0.6)	3	2	3	4	3
Q16	The RHO generates sufficient force while opening.	3.6 (0.5)	3	4	3	4	4
Q17	The RHO opens and closes quick enough.	4.2 (0.7)	5	3	4	5	4
Q18	The RHO is lightweight enough.	4.4 (0.5)	5	4	4	5	4
Q19	The RHO is well and firmly attached to the hand.	3.4 (1.0)	3	2	3	5	4
Q20	The movement generated by the RHO is pleasant.	4.0 (0.6)	4	4	3	5	2
	Total score $CUQ_{total}$	63.5 (16.0)	77.5	57.5	35.0	77.5	70

Individual ratings and mean and standard deviation for each item rating and the overall scores are reported of the participants of group B

Individual ratings range from 1 (completely disagree) to 5 (completely agree), and total score ranges from 0 to 100, wherein a higher value corresponds to a better rating of the RHO. (*: group B; SD: standard deviation)

#### Qualitative findings

We collected over 100 user statements from the semi-structured interviews and from spontaneous thinking aloud during the testing sessions. Statements ranged from general user experience comments to wishes and improvement suggestions specifically related to the tested hardware and software. In addition to the five core attributes (i.e., *functional benefit*, *device functionality*, *ease of use & practicability*, *comfort*, and *safety*), five additional attributes were introduced during the thematic analysis (i.e., *aesthetics*, *desirability*, *learnability*, *adaptability & customization*, and *complexity*) and all qualitative findings were assigned to one of the resulting attributes.

The complete thematic analysis of statements and observations and their assignment to specific usability attributes is provided in Additional file 2: File 2.

#### Adverse events and technical issues

We reported no adverse events during the study sessions. No injuries, such as cuts or bruises, were caused by wearing the RHO, and none of the sessions had to be discontinued due to adverse events. In eight subjects, minor temporary pressure marks from the textile strap at the lower arm or from the tight elastics of the glove attachment were observed and reported. One participant (P6) realized that the muscle tone for her functional, passive grasp (tenodesis grasp) was decreased due to the unusual but painless stretching through RHO extension and flexion after session 1. This effect lasted for almost two weeks and eventually led to a painful feeling of overstretching, which had to be checked by a medical professional. The previous, chronic tone and functionality of the passive grasp returned eventually, with no additional impairment or pain residing. The study coordinators agreed with the participant to discontinue the study to avoid potential negative impacts on the participant’s hand function and ability to perform daily life activities.

Assessing the device functionality in terms of technical robustness, we could observe that the intense usage of the RHO triggered both minor and major technical failures. Minor issues included a loosening of components or the need to substitute straps, which could be resolved on the spot. Three major technical failures (each occurred once) prevented the functional use of the RHO and thus led to session interruptions and rescheduling: (i) failures related to the actuation system (torn Bowden cable), (ii) the low-level control (error in motor control signal), (iii) and the mechanics of the hand module (insufficient motion due to blocked mechanism).

## Discussion

The short-term usability of the RELab tenoexo, an RHO to assist hand function in people with SCI, was evaluated with a mixed methods approach. With a total of 15 users, the RHO was evaluated in a study design employing both qualitative and quantitative methods to holistically assess usability. We discuss our main findings in dedicated sections focusing on specific usability attributes to provide a comprehensive review of the RELab tenoexo and generate generalizable insights relevant for the comparison with similar RHO.

### Immediate functional benefit for people with severe hand impairment

A mean improvement in hand function above the clinically important difference on the ARAT [33] was found in group B, with three participants achieving at least 9 additional points and one being slightly below clinically important difference. This underlines a significant immediate functional benefit from the active assistance of the RELab tenoexo. An improvement in the “Grasp” and the “Grip” subscale of the ARAT could be observed for all participants of group B, while most participants’ scores in the “Pinch” stayed similar or even decreased with the RHO. This can be explained by the functional principle of the RELab tenoexo, as it supports mainly gross grasping motion and provides additional grip strength rather than assisting dexterity. Similar observations were made by Yun et al. [35] and Radder et al. [36] in their evaluations of RHO for target users with hand impairments. In both studies, participants also faced difficulties in fine motor tasks while achieving higher function in tasks requiring a power grasp when using the respective RHO. The decreased ARAT score observed for one of our study participants (P13, − 10 points), compared to the unassisted condition, further highlights this limitation of the RELab tenoexo design. Without the RHO, the participant was able to pinch small objects, as well as lift and manipulate larger objects using a tenodesis grasp and compensatory movements (indicated also by the highest GRASSP score in group B). With the current design of the RHO and without a specific personalization in terms of device size and fit, these strategies were restricted such that the ARAT score was lower with the device.

Four participants of group A discontinued the study due to too weak proximal upper limb function. These insights indicate that the RELab tenoexo is most beneficial for people with more severe distal (hand) impairment, low tone, and residual proximal upper limb function. It is yet to be evaluated if combining the RHO with passive gravity compensation mechanisms or wearable robotic devices for the proximal joints could widen the target population. Similarly sleek and lightweight solutions such as by Georgarakis et al. [37] or O’Neill et al. [38] could combine well with the portable design of the RELab tenoexo.

Furthermore, the RELab tenoexo works best with persons that experience a flaccid paralysis of the hand. For individuals with a strongly increased muscle tone or spasticity in the hand muscles, the extension force provided by the RELab tenoexo is not sufficient to open the fingers to a fully extended position. These findings have been further demonstrated and supported in a device variation tailored to the pediatric population [39].

The subjective data from the SUS and CUQ correlates with the ARAT scores, as participants rated the usability of the RHO higher if there was a clearer functional benefit and vice-versa. More specifically, the participants of group B agreed that they would like to use the RHO frequently, mainly because the device enabled them to perform the tasks more easily, quickly, and with more voluntary control of their hand function.

### The RELab tenoexo is perceived as comfortable, ergonomic, and safe

Among other aspects, we specifically studied the human-robot interaction in terms of comfort, ergonomics, and safety with a range of outcome measures in this work. First and foremost, no major discomfort required a premature termination of any testing session during a total usage time (i.e., sum over all participants) of more than seven hours (RHO powered, donned). In terms of ergonomics, as rated by dedicated CUQ items, the RHO weight was considered adequate (Q18), the closing and opening speed were good (Q17), and the guided motion was comfortable (Q20). The dimensions and weight of the RELab tenoexo also fulfill the RHO design criteria defined by Boser et al. [23], who established RHO requirements from the perspectives of target users with hand impairments and clinicians. Further, we can infer from the gross movement subscale of the ARAT that the weight and dimensions of the RHO are nonrestrictive, as all participants were able to achieve the same freedom of motion with and without the RHO. Still, a few participant statements were in favor of a further reduction in weight (*“Less weight would improve handiness, especially when wearing but not using the RHO.”*)

The absence of major adverse events arguably indicated the general physical RHO safety. The individual adverse effect of overstretching for one participant derives from the physiological shortening/contraction in hand muscles and tendons after years of reduced use. In these cases, a RHO to force hand flexion and extension is not a viable solution to assist hand function due to the risk of injuries and the restriction of trained compensatory movements (e.g., tenodesis grasp) that could better support ADL. The current fixed-wrist design of the RELab tenoexo restricts trained compensation movements such as the tenodesis grasp and increases the finger rigidity in a flexed position, requiring a higher force from the RHO to extend the fingers. A more adjustable or flexible wrist design as proposed by Dittli et al. [40] could allow for more versatile support from the RHO.

### Donning is key for ease of use and device adoption

While ergonomics and safety are absolute must-have criteria for any wearable robotic device, the ease of use is arguably one of the most influencing factors determining actual daily use and most frequently related to overall usability [4, 41–43].

The ease of use was assessed subjectively by the SUS (Q3, Q4, Q8) and objectively by the donning time. In combination with our qualitative results, we could assess the ease of use of the RHO in two dedicated usage phases: (i) preparation and donning and (ii) active operation.

While the overall ease of use was rated positively, lower ratings of SUS items Q4 and Q8, CUQ item Q14, and user feedback from the semi-structured interviews indicated that the donning process (i), which required assistance, was considered cumbersome and a limitation to frequent and independent daily use (*“It is a disadvantage that I can not don the RHO independently.”*). The observation that the donning process and physical attachment system are critical to device adoption has also been made in other studies [13, 36, 39, 44, 45]. However, the donning interface and process of RHO is rarely reported and analyzed in detail. Based on the results of this study, the overall complexity of RHO (i.e., number of components and donning steps) and the donning time should be reduced to increase the ease of use and intuitiveness, indicated by the high rating of SUS item Q2 and subjective feedback, respectively. An average donning time of 4 min 55 s was recorded across all testing sessions when performed at an eased pace. Similar donning times were recorded in a previous clinical utility study with adapted version of the RELab tenoexo for pediatric target users [39] and a dedicated usability study investigating the manual-guided setup of the RELab tenoexo on a mock-user (neurologically intact hand) by untrained persons in a caregiver role [41]. Accordingly, a donning time of approximately 300 s is the current benchmark for the RELab tenoexo. Yurkewich et al. [44] found that an average donning time of 180 s for their RHO was perceived as cumbersome, indicated by a low rating of ease of use in the QUEST usability questionnaire. Accordingly, target users seem to expect this process to be done more efficiently, especially when performed by an assisting helper. Most participants stated that optimally, they would be able to don the device by themselves, imposing a major challenge, particularly for glove-based systems intended for users with bilateral hand impairment. Radder et al. reported further usability limitations that need to be considered in the design of the physical attachment system for RHO, such as heat building and sweating [36]. In the semi-structured interviews, many participants provided valuable suggestions to improve the physical attachment system of the RHO to fit their individual needs better (*“A donning aid would be helpful.”*).

In terms of active device operation (ii), participants were provided push buttons as the only option to trigger RHO motion. While other input modalities such as electromyography (EMG) [35] or force myography [46] may provide more natural, direct control for RHO users [45], buttons appear to offer the most robust and simple solution as of today [4]. All participants quickly learned how to operate the RHO with the button, as reflected by the high ratings in SUS items Q7 and Q10. In the semi-structured interview that followed the second session, several participants agreed that a selection of additional intention detection systems (e.g., voice control or a smartphone application) could further increase the RHO usability.

Other phases of the usage cycle such as doffing, storage, or maintenance (e.g., cleaning and disinfecting, recharging of batteries) were not specifically investigated in this study, although being an integral part of potential daily use. For future studies, these aspects should be considered in the evaluation of ease of use.

### Individual needs call for tailored solutions

One of the main observations and learnings of our study sessions was the astonishing diversity of needs (and wishes) of our participant sample. Within our group of users with very similar impairment types (e.g., GRASSP score, injury level, time-post-injury), not all would be willing and able to use the RELab tenoexo due to their strongly differing personal and environmental context (e.g., expectations, living situation, previous experiences). As for any assistive device, the match between person and technology has to be carefully evaluated and optimized in order to achieve technology acceptance [47]. In the case of RHO, tailoring could solve usability issues with functional benefit and comfort that were identified in our analysis, and improve the match to the individual target user needs. Our results indicate that critical aspects which should be tailored to the user are device size (*“I would need a more flexible adaptation to hand size”*), aesthetics (*“The RHO looks sleek.”*; *“I don’t like the looks of the RHO”*), device functionality (*“It really feels like a firm grasp.”*; *“Fingertips should close more.”*), and user interfaces. In particular, the limited size availability and adjustability of the RELab tenoexo led to the discontinuation of three participants and may limit the ergonomics, user comfort, and safety (e.g., due to misalignment in the force transmission to the hand). We thus suggest that, if possible, RHO should be specifically tailored for individual users instead of aiming for a one-fits-all concept. Tailoring of a RHO could be achieved with modular systems, providing a range of solutions from which the user can choose from (e.g., physical attachment systems, different combinations of intention detection systems).

### Gained value from the mixed methods approach

The most important contribution of the mixed methods approach is likely the large number and variety of qualitative user statements collected from two sessions in this work (e.g., improvement suggestions, critical feedback) and the rich data to understand if the targeted problem has been appropriately addressed. The quantitative outcome measures largely covered the predefined core attributes. However, the thematic analysis showed that these attributes were not sufficiently broad to cover all aspects of usability. Thus, the analysis uncovered five additional attributes relevant to the target users. These allowed us to gain important insights into the user’s perception of the usability of the RHO (e.g., in terms of aesthetics, complexity, or learnability), which would have been missed in an evaluation using purely quantitative measures.

The value of mixed method evaluations and thematic analyses has been highlighted before for other rehabilitation devices than RHO. Bhattacharjya et al. [19] and Warland et al. [18] used a mixed methods approach (e.g., quantitative surveys, semi-structured interviews) to evaluate devices for upper limb therapy after stroke. They claim that the approach allowed capturing a holistic picture of the system usability [18] and that the qualitative findings strengthened the validity of quantitative findings or, when different, provided possible explanations for the results found [19]. In both studies, the qualitative data were analyzed regarding specific device attributes rather than general usability attributes. We believe that our analysis based on generalizable usability attributes extracted from literature and identified during post-processing enables more directly transferable results to other RHO.

### Design limitations of the RELab tenoexo and improvement suggestions

Besides the general insights relevant for other RHO designs, residual usability limitations specific to the evaluated RELab tenoexo were observed that need to be addressed in the next design iteration.

Even though the majority of our study participants appreciated the grasp strength provided by the RELab tenoexo, a further increased grasp force would be desirable, as indicated by CUQ items Q15 and Q16, as well as by the discontinuation of two participants due to insufficient opening/closing force for spastic hands. Increasing the provided assistive force in both flexion and extension could enlarge the target user group and further increase the functional benefit from the RHO. However, an excessively increased force on the users hands may negatively impact the ergonomics and comfort of the RHO, in which case alternative solutions such as reduction of spasticity via medication could be investigated. Other design features of the RHO, such as the thumb opposition mechanism and the fixed wrist position, might need to be refined and optimized for individual needs and capabilities. Adjusting the thumb opposition should require less effort (e.g., by increasing the handle size or adding a loop to it). The wrist should be adjustable in position or flexible not to restrain residual function (e.g., wrist extension for a tenodesis grasp).

Several robustness issues resulting in technical failures of the actuation system, control, and mechanics of the hand module were recorded. The RELab tenoexo hardware was also used for other studies in parallel, which might have increased the wear of the device and thus prevented drawing a conclusion as to how the use recorded in this study led to the technical issues. However, to enable long-term use of the RHO in potentially unsupervised settings, these failures have to be further investigated and addressed.

### Methodological considerations and limitations

In this study, we evaluated the short-term usability as only one step of an iterative design process. Therefore, it cannot be concluded whether the selected approach generates a concrete benefit and whether the identified usability issues can be successfully addressed in the next design iteration. Further, a learning effect on the RHO might influence some aspects of usability such as the functional benefit and easy of use. A similar protocol should be optimally conducted repeatedly after completed design iterations to optimally follow a user-centered design approach. Also, further testing in the intended usage environment (i.e., outside of the controlled laboratory environment) will be required to investigate the device uptake by the target users as well as other technology stakeholders. Yet, the comprehensive mixed methods evaluation applied in this study allowed us to capture a broad picture of the short-term usability of the RELab tenoexo and identify directions for future developments. The presented approach to usability evaluation could potentially be applied for other RHO with minor adaptations of the protocol to specific evaluation focuses and target user groups. Such studies could help to challenge insights of this study and identify global trends and gaps in RHO development.

Even though the assessment with the ARAT generated valuable insights, it is important to state that the clinical test has not been validated for individuals with an SCI. Further, the ARAT does not evaluate bimanual tasks, while most daily activities require the use of both hands simultaneously. Alternatively, more SCI-specific hand function tests such as the JTHFT or the TRI-HFT or assessments involving bimanual tasks such as the BeBiTT [48] would allow for more reliable characterization of hand function. Furthermore, results from interviews and think-aloud methods collecting semi- or unstructured user statements have to be interpreted with care, as they are prone to primarily capture usability issues due to the human’s natural negativity bias [49]. Users are more likely to point to things they think can be improved rather than things they find good and satisfying. Lastly, our analysis might be biased due to the small number of participants of group B or by statements of individual participants if others did not comment on specific attributes. Particularly qualitative evaluations are often biased by several personal factors such as expectations or learning effects, which may have influenced, e.g., the decrease in SUS scores from session 1 to session 2 in some participants in this study. The best way to mitigate these effects would be to have multiple evaluation sessions and more users to reduce potential bias in overall results due to individual users’ opinions from single sessions.

## Conclusion

We evaluated the short-term usability of the RELab tenoexo using a mixed methods approach. RELab tenoexo provided an immediate improvement in hand function and received high acceptance from individuals with strong hand impairment after SCI. The detailed report of usability attributes affecting RHO usability may be of value for other researchers in the field. Future developments of RHO and assistive devices in general should focus on increasing the ease of use and the tailorability and adaptability to meet the largely varying needs and demands of individual users. Particular attention should be given to factors influencing device usability identified in this work, such as the donning process and physical attachment system. A mixed methods approach as proposed in this paper might be generalizable to other RHO. Further detailed reports of usability evaluations in the intended environments of RHO could generate more holistic and transparent reviews of current technologies, provide valuable insights for other researchers in the field, and accelerate the process toward the integration of technology into the everyday life of people with sensorimotor impairment.

### Supplementary Information

**Additional file 1:** **File 1.** Semi-structured interview questions. **File 2. **Thematic analysis of quantitative and qualitative data.

## Data Availability

The data sets generated and/or analyzed during the current study are not publicly available.

## Abbreviations

ARAT: Action research arm test
CUQ: Custom usability questionnaire
EMG: Electromyography
JTHFT: Jebsen-Taylor hand function test
RAS: Remote actuation system
RHO: Robotic hand orthosis
SCI: Spinal cord injury
SD: Standard deviation
SUS: System usability scale
TRI-HFT: Toronto rehabilitation institute hand function test
UCD: User-centered design
