# Supplementary material for: Mixed methods usability evaluation of an assistive wearable robotic hand orthosis for people with spinal cord injury
Source: J Neuroeng Rehabil. 2023 Dec 1;20:162. doi: 10.1186/s12984-023-01284-8 (PMC10693050; doi:10.1186/s12984-023-01284-8)
Supplement: Supplementary file 1 — Additional file 1: File 1. Semi-structured interview questions. File 2. Thematic analysis of quantitative and qualitative data. [file 12984_2023_1284_MOESM1_ESM.pdf]

Additional Files

Additional file 1 — Semi-structured interview questions

Rehabilitation status

Q21: Are you currently undergoing therapy of the upper extremities? If so, what kind of therapy?

Q22: Do you have experience with robot-assisted rehabilitation? If so, please describe the therapy.

Q23: Have you used / do you use assistive devices? If so, please describe these devices. (active/passive?)

Q24: What do you use your current assistive device for?

Q25: For which activities and how long do you use your current assistive device? (in everyday life, for therapy, etc.)

Q26: Why did you choose this assistive device?

Q27: For chronic patients: Which everyday situations can you manage well with your hands and for which do you need help or an aid?

Unmet Needs and Wishes

Q28: What activities do you want to have an assistive device for? (Tasks: Personal hygiene, writing/Objects: Bottle, pen)

Q29: In which situations would you use this assistive device? (at home, at work, in public, in therapy)

Q30: How often would you like to use this assistive device? (whole day, sporadically, in specific situations)

RHO User Experience

Q31: What did you like about tenoexo? What was the most positive aspect? (design, components, weight, etc.)

Q32: What did you not like about tenoexo? What was the most negative aspect? (design, components, weight, etc.)

Q33: In which situations do you think you would use the tenoexo? (at home, at work, in public, in therapy)

Q34: How often do you think you would use the tenoexo? (whole day, sporadically, in specific situations)

Q35: How would you want to control the tenoexo? (Buttons, muscular signals, brain signals, . . .)

Q36: Where would you like to place the control unit? (integrated in the wheelchair, independent of the wheelchair)

Q37: Do you feel any pressure marks on your hand? If so, please describe the location.

Q38: Can you move your hand together with the tenoexo as freely as without? If no, to what extend are you restricted?

Additional file 2 — Thematic analysis of quantitative and qualitative data

Table 5 contains the thematic analysis of quantitative and qualitative data. More general insights and statements not related to the RELab tenoexo but RHO or robotic assistive technologies in general were not assigned to a specific attribute, but added to the last row of the table.

Table 5: **Thematic analysis of quantitative and qualitative data:** + = core attributes, Q_i = quantitative questionnaire item, SSI = semi-structured interview, TA = thinking aloud, O = user observation notes, *italic text* = exemplary anecdotal feedback, N.A.: not applicable.

Attribute	Quantitative Findings	Qualitative Findings	Source	Ocurrence
Functional benefit+	· ARAT	· **Increase of grasp strength**	**SSI**	**N=6**
	· SUS (Q1)	· **Insufficient grasp strength**	**SSI**	**N=6**
	· CUQ (Q11,Q12,Q13)	· **Confidence in grasp**	**SSI,TA**	**N=2**
		· *"It really feels like a firm grasp."*	SSI	
		· *"Additional grip provided by glove (non-slippery surface) is helpful."*	TA	
Device functionality+	· ARAT	· **Insufficient finger range of motion**	**SSI**	**N=4**
	· SUS (Q1,Q5,Q6)	· *"Unfortunately, I can not fully close my hand to a fist."*	SSI	
	· CUQ (Q15,Q16,Q17)	· *"I would want to move fingers individually."*	SSI	
	· Adverse events	· *"Fingertips should close more."*	SSI	
	· Technical issues	· *"My fingers flex less than without RHO."*	SSI	
		· **Wrist movement stabilized by hand module**	**SSI**	**N=2**
		· **Wrist movement restricted by hand module**	**SSI**	**N=1**
		·*Fixed wrist position restricts tenodesis grasp."*	SSI	

Table 5: **Thematic analysis of quantitative and qualitative data - continued:** $^{+}$ = core attributes, Q_i = quantitative questionnaire item, SSI = semi-structured interview, TA = thinking aloud, O = user observations notes, *italic text* = exemplary anecdotal feedback, N.A.: not applicable.

Attribute	Quantitative Findings	Qualitative Findings	Source	Occurrence
Ease of use & practicability^{+}	· SUS (Q3,Q4,Q8)	· **Difficulties in donning and doffing**	**SSI,TA**	**N=9**
	· CUQ (Q14)	· *"The main issue is the donning of the glove and the attachment of the hand module."*	TA	
	· Donning time	· **Importance of independent donning and doffing**	**TA**	**N=2**
		· *"It is a disadvantage that I can not don the RHO independently."*	TA	
		· *"I can not imagine somebody helping me to don it at home."*	TA	
		· **Suggestions to simplify donning**	**TA**	**N=3**
		· *"Magnets or leashes around the fingers instead of Velcro would be better."*	TA	
		· *"Use separate small straps instead of a glove to fix the hand module to the hand."*	TA	
		· *"A donning aid would be helpful."*	TA	
		· **Inability to manually oppose thumb**	**TA,O**	**N.A.**
		· Thumb opposition was possible for some, but not all users.	O	
		· *"The thumb should be easier to move."*	TA	
		· **Approval of movement timing**	**TA**	**N=1**
		· *"The delay between trigger and motion was helpful to reposition the hand towards the object."*	TA	
		· **Approval of button as intention detection strategy**	**SSI,TA**	**N=6**
		· *"Using the buttons to control the RHO is very useful and easy to use."*	TA	
		· **Openness to other intention detection strategies**	**SSI**	**N.A.**
		· Open to use electric muscle signals.	SSI	N=2
		· Open to use voice control.	SSI	N=4
		· Open to use brain signals.	SSI	N=2
		· Open to use a foot pedal.	SSI	N=1
		· Open to use a smartphone.	SSI	N=2
		· **Preferred placement of back module**	**SSI**	**N.A.**
		· Attached to wheelchair.	SSI	N=7
		· Attached to back.	SSI	N=1
		· Attached to upper arm.	SSI	N=1

Table 5: **Thematic analysis of quantitative and qualitative data - continued:** $^{+}$ = core attributes, Q_i = quantitative questionnaire item, SSI = semi-structured interview, TA = thinking aloud, O = user observations notes, *italic text* = exemplary anecdotal feedback, N.A.: not applicable.

Attribute	Quantitative Findings	Qualitative Findings	Source	Occurrence
Comfort & ergonomics^{+}	· ARAT	· **Approval of hand module weight**	**SSI**	**N=3**
	· CUQ (Q18,Q19,Q20)	· **Need for reduction of hand module weight**	**SSI**	**N=1**
	· Donning time	· *"Less weight would improve handiness, especially when wearing but not using the RHO."*	SSI	
		· **Adequate comfort of glove**	**SSI**	**N=1**
		· *"The glove is comfortable. It is like a normal glove"*.	SSI	
		· **Inadequate comfort of glove**	**TA,O**	**N=5**
		· *"The glove is too warm."*	TA	
		· Inadequate size of glove	O	
		· **Inadequate fit of hand module**	**SSI,O**	**N=4**
		· Uncomfortable pressure on thumb	SSI	
		· Fingers of RHO were too long	O	
		· Thumb position too distal or proximal	O	
		· **Minor pressure marks**	**O**	**N.A.**
		· Wrist	O	N=3
		· Metacarpophalangeal joints	O	N=1
		· Little (pinky) finger	O	N=1
		· Thumb	O	N=1
		· Fingertips	O	N=5
		· **Suggestions to improve comfort**	**TA**	**N=1**
		· *"More material between RHO and hand would be more comfortable."*	TA	
		· **No restriction of upper-limb workspace**	**SSI**	**N=3**
		· **Restriction of upper-limb workspace**	**SSI**	**N=3**
		· *"The system is unhandy."*	SSI	
		· *"The cables between backpack and hand module restricted my hand movement a bit."*	SSI	
Safety^{+}	· SUS (Q9)	· **Safety concerns**	**SSI**	**N=1**
	· Adverse events	· *"Something might get stuck in the open mechanics of the finger mechanism."*	SSI	

Table 5: **Thematic analysis of quantitative and qualitative data - continued:** $^{+}$ = core attributes, Q_i = quantitative questionnaire item, SSI = semi-structured interview, TA = thinking aloud, O = user observations notes, *italic text* = exemplary anecdotal feedback, N.A.: not applicable.

Attribute	Quantitative Findings	Qualitative Findings	Source	Occurrence
Aesthetics	N.A.	· **Approval of appearance of RHO**	**SSI**	**N=1**
		· *"The RHO looks sleek."*	SSI	
		· **Disapproval of appearance of RHO**	**SSI**	**N=1**
		· *"I don't like the looks of the RHO."*	SSI	
		· **Relevance of appearance**	**SSI**	**N=2**
		· *"I don't want to raise attention if wearing the RHO in public."*	SSI	
		· *"Looks don't matter."*	SSI	
		· *"I would rather look like a cyborg to being unable to do anything independently."*	SSI	
Desirability	· SUS (Q1)	· **Frequency of desired use**	**TA**	**N=1**
		· *"Depends on functionality and usability, including aesthetics, weight, and robustness."*	TA	
Learnability	· ARAT	· **Need for training**	**TA,O**	**N.A**
	· SUS (Q7, Q10)	· *"It needs a lot of practice until one can use it."*	TA	
		· *"I would need more adaptation time to use it in daily life."*	TA	
		· Sometimes a few attempts required to figure out the positioning of the hand relative to the object.	O	
Adaptability & customization	N.A.	· **Need for hand size adaptability**	**SSI,O**	**N.A.**
		· *"It would need a more flexible adaptation to hand size"*	SSI	
		· Some subject's hands were strongly swollen, which can occur temporarily.	O	
Complexity	· SUS (Q2,Q4)	· **Number of components too high**	**SSI,TA**	**N=3**
		· *"There are too many cables."*	SSI	
		· *"The system has too many components and steps required to set it up to be useful."*	TA	
		· *"If everything was integrated in one glove, it would be awesome."*	TA	
		· **Need for Simplicity**	**TA**	**N=1**
		· *"The system should be kept as simple as possible, especially for donning."*	TA	

Table 5: **Thematic analysis of quantitative and qualitative data - continued:** $^{+}$ = core attributes, Q_i = quantitative questionnaire item, SSI = semi-structured interview, TA = thinking aloud, O = user observations notes, *italic text* = exemplary anecdotal feedback, N.A.: not applicable.

Attribute	Quantitative Findings	Qualitative Findings	Source	Occurrence
Findings related to general RHO or assistive devices	N.A.	· **Assistive device need**	**SSI**	**N.A.**
		· Large variety of activities of daily living (ADL) in which an additional assistive device is needed.	SSI	
		· **Compatibility and added benefit**	**SSI**	**N.A.**
		· Passive orthoses already used for wheelchair propulsion or dedicated tasks, e.g., writing or eating.	SSI	
		· An RHO should replace passive orthoses or not restrict their added benefit.	SSI	
		· **Potential RHO use cases**	**SSI**	**N.A.**
		· Primary use for eating and drinking or preparing food.	SSI	N=4
		· At home, for work, or leisure activities like gardening or horseback riding.	SSI	
		· **Reasons for use of other assistive devices**	**SSI**	**N.A.**
		· Because they were recommended by their therapist.	SSI	N=7
